# Supplementary material for: A crab swarm at an ecological hotspot: patchiness and population density from AUV observations at a coastal, tropical seamount
Source: PeerJ. 2016 Apr 12;4:e1770. doi: 10.7717/peerj.1770 (PMC4841253; doi:10.7717/peerj.1770)
Supplement: Table S1 [file peerj-04-1770-s002.pdf]

Supplemental Materials, Table 1. Mass stranding events of *Pleuroncodes planipes* in Southern California from January to August 2015 documented by multiple media outlets.

**Date of observation**	**Publication Date**	**Reported Location(s)**	**Source**	**Author**	**Article Title**
1/21/15	1/22/15	Newport Beach	Los Angeles Times	Hannah Fry	Thousands of fiery red crabs wash ashore in Newport Beach
2/21/15	2/21/15	Balboa Island, Newport Beach	Orange County Register (California)	Laylan Connelly	Thousands of small crabs wash up on O.C. coast in Balboa
6/14/15	6/15/15	Dana Point, San Clemente, south Laguna, Newport Beach, Huntington Beach	Orange County Register (California)	Laylan Connelly	Red crabs blanket O.C. beaches
6/14/15	6/15/15	Orange and San Diego Counties	Los Angeles Times	Bryce Alderton	Thousands of tiny red crabs invade Southern California beaches
6/14/15	6/17/15	San Pedro, Rancho Palos Verde	Daily Breeze	Megan Barnes	Tiny red crabs wash up on beaches in San Pedro and Rancho Palos Verdes
6/14/15	6/17/15	Seal Beach to San Clemente	Orange County Register (California)	Laylan Connelly	They're dead. They're red. They're smelly. (No, you can't eat them.)
6/14/15	6/19/15	Laguna Beach, Huntington Beach, Newport Beach (Huntington Beach to San Clemente)	Laguna Beach Coastline Pilot (Costa Mesa, California)	Bryce Alderton	Red crabs cover O.C. beaches from H.B. to San Clemente
6/12/15	6/19/15	San Diego Sunset Cliffs	Los Angeles Times	Christopher Reynolds	Video: What does the red tide at San Diego's Sunset Cliffs portend?
8/15/15	8/28/15	Channel Islands	Los Angeles Times	Veronica Rocha	Watch thousands of tiny red crabs invade Channel Islands

Supplemental Materials, Video S1. *Pleuroncodes planipes* filmed from the M/V Alucias's RV2 submarine. (Video file uploaded separately, under the name "*Hannibal_swarm_by_Pineda_subm.mp4*"
